# Temperamental and Character Traits as Risk Factors for Binge Eating Disorder in Women with Polycystic Ovary Syndrome

**DOI:** 10.3390/jcm13237100

**Published:** 2024-11-24

**Authors:** Katarzyna Suchta, Roman Smolarczyk, Marta Hajbos, Andrzej Kokoszka

**Affiliations:** 1 Department of Gynecological Endocrinology, Medical University of Warsaw, 00-315 Warsaw, Poland; 2 II Department of Psychiatry, Medical University of Warsaw, 02-091 Warsaw, Poland

**Keywords:** PCOS, binge eating disorder, temperament, character, personality

## Abstract

**Background/Objectives**: Polycystic ovary syndrome (PCOS) is one of the most common endocrine disorders in women of reproductive age. It is associated with an increased risk of somatic and mental health problems. The prevalence of binge eating disorder (BED) in women with PCOS is higher than in the healthy population. The co-occurrence of PCOS and BED increases the risk of negative health outcomes and may worsen patient compliance. For both groups of disorders, specific personality traits have been described in the literature. The aim of this study was to assess temperament and character in PCOS women with and without BED, in search of specific personality traits that may distinguish PCOS patients with a higher predisposition to BED. **Methods**: 128 women diagnosed with PCOS according to the modified Rotterdam criteria were included in the study. All completed the Temperament and Character Inventory by R.C. Cloninger (TCI). The collected data were statistically analyzed. **Results**: The PCOS–BED subgroup was characterized by specific personality traits in comparison with PCOS subgroup: statistically significantly higher scores of harm-avoidance dimensions (in anticipatory worry, shyness, and fatigability subscales) and statistically significantly lower scores of persistence and self-directedness dimensions (in purposeful, resourcefulness, self-acceptance, and enlightened second nature subscales). **Conclusions**: PCOS–BED women have certain personality traits. Screening PCOS patients for the presence of certain personality traits could identify those at risk. PCOS–BED women may be at higher risk of health problems and treatment failure and may require a different therapeutic approach to treatment, including psychotherapeutic interventions.

## 1. Introduction

Polycystic ovary syndrome (PCOS) is one of the most common endocrine disorders in women of reproductive age. It is diagnosed on the basis of the modified Rotterdam criteria, which include the following: clinical and/or biochemical hyperandrogenism; anovulatory menstrual cycles and/or infrequent ovulations; and a characteristic ovarian structure on ultrasound examination [1].

This endocrine disorder has many negative effects on women’s health. It is known to increase the risk of infertility, menstrual disorders, and cardiometabolic conditions such as obesity, arterial hypertension, impaired glucose tolerance, impaired fasting glucose, hyperinsulinemia, or type 2 diabetes. The results of a meta-analysis suggest that almost one in two women with PCOS is obese [2].

Interestingly, both obese and lean women with PCOS have higher levels of visceral adipose tissue compared to healthy controls. For this reason, women diagnosed with PCOS are at increased risk of metabolic consequences [3]. Women with PCOS are also more likely to be diagnosed with autoimmune thyroid disease, mental health problems, and eating disorders. The diagnosis of PCOS has an impact on both the somatic and mental well-being of women, and the frequency of its diagnosis makes it a challenge for the healthcare system.

According to the current Diagnostic and Statistical Manual of Mental Disorders (DSM-5), binge eating disorder (BED) is characterized by recurrent binge eating episodes that have occurred at least once a week for three months and are associated with marked distress [4].

Many studies have shown that the prevalence of BED in women with PCOS is higher than in the general population, ranging from 12% to 42% [5,6,7]. The presence of BED is known to increase the risk of obesity-related health complications such as certain cancers, diabetes, hypertension, heart disease, stroke, sleep problems/disorders, and asthma and gastrointestinal symptoms and disorders, menstrual dysfunction, pregnancy complications, intracranial hypertension, and pain conditions. People with BED also have an increased risk of mortality. It can also lead to deterioration in mental health. In women with PCOS, binge eating disorder can influence and worsen menstrual disorders, pregnancy complications, and hyperandrogenism and worsen treatment outcomes [8,9,10,11].

Given the increased prevalence of BED in PCOS patients, the exacerbation of health complications in PCOS patients with coexisting BED, and the poorer response to standard treatment, the authors believe it is crucial to find prognostic criteria in PCOS patients that can help identify individuals at higher risk of having BED.

There are many studies in the literature that indicate specific personality traits in PCOS patients. For example, Ozcan Dag Z. et al. indicate that the patients with PCOS had significantly higher rates of depressive, anxious, and hyperthymic scores compared to controls [12].

In his study, Urban W. et al. points out that PCOS patients have more pronounced characteristics of Type D personality and have higher neuroticism and lower openness to experience and conscientiousness [13].

On the other hand, patients with eating disorders are also found to have specific personality traits. Fassino S. et al. indicate that patients with binge eating disorder differ from controls in novelty seeking, harm avoidance, cooperativeness, and self-directedness. The author points out that the self-directedness dimension seems to be the stronger predictor of BED [14].

Dalle Grave R. et al. also point out in their study that patients with binge eating disorder have higher scores on the novelty-seeking and harm-avoidance dimensions and lower scores on the self-directedness dimension [15].

Favaretto E, et al. [16] summarize research from the last 30 years on how certain temperamental traits may contribute to mental disorders. In the literature, certain temperamental profiles are associated with the risk of particular psychiatric and somatic disorders. Temperamental dimensions such as harm avoidance are related to behavioral outcomes in clinical populations. Alterations in this dimension may be observed in individuals with cyclothymic or depressive personality traits. Both of these temperamental traits are associated with a higher risk of eating disorders and obesity. The results of the study support the concept that assessing personality in women with PCOS may be helpful in identifying individuals at higher risk of binge eating disorder.

To the best of our knowledge, there is no study on the personality characteristics of PCOS women with a co-occurrence of binge eating disorder. We believe that identifying characteristics that could be used as predictors of the occurrence of binge eating disorder in PCOS patients would be helpful in choosing appropriate treatment that includes behavioral intervention, psychoeducational interventions, psychotherapy (mainly cognitive behavioral therapies—CBT; dialectic behavioral therapy—DBT; or interpersonal psychotherapy—IPT) and improve compliance and effects of therapy in this group of women.

The aim of this study is to assess the temperament and character of PCOS women with and without BED, in search of specific personality traits that may distinguish PCOS patients with a higher predisposition to BED.

## 2. Materials and Methods

### 2.1. Ethics Statement

The study was approved by the Ethics Committee of the Medical University of Warsaw. All patients included in the study were Polish Caucasians attending the Endocrinology and Gynaecology Clinic of the Department of Endocrinology and Gynaecology of the Medical University of Warsaw. Patients gave written informed consent before participating in the study.

### 2.2. Study Population

A total of 128 women diagnosed with PCOS, aged 16 to 46 years, were recruited for this study. The study group was assessed for binge eating disorder. Temperament and personality were also assessed. The study group was then divided into three subgroups:

- Group 1—PCOS women without binge eating disorder (47 women) [PCOS],
- Group 2—PCOS women who did not meet the diagnosis of binge eating disorder but answered positively for at least one question when evaluated for DSM-5 criteria of binge eating (27 women) [PCOS–non-BED]
- Group 3—PCOS women with diagnosis of binge eating disorder (54 women) [PCOS–BED].

#### 2.2.1. PCOS Assessment

The diagnosis of PCOS was made using the Rotterdam criteria, modified by international consensus from the American Society for Reproductive Medicine (ASRM), the Endocrine Society (ENDO), the European Society of Endocrinology (ESE), and the European Society of Human Reproduction and Embryology (ESHRE). The criteria include clinical and/or biochemical hyperandrogenism; anovulatory menstrual cycles and/or infrequent ovulations; and characteristic ovarian structure on ultrasound [1]. A description of the criteria used in the study are in supplementary materials in Table S1.

Exclusion criteria were patient refusal to participate in the study and a diagnosis of hyperandrogenism other than PCOS, such as non-classical adrenal hyperplasia, androgen-secreting tumor, Cushing’s syndrome, hyperprolactinemia, or non-euthyroid thyroid dysfunction. An additional exclusion criterion was recent pregnancy, use of oral contraceptives, glucocorticoids, biguanides, or GLP-1 receptor agonists for up to six months prior to enrolment, as all these medications can affect hormonal and biochemical parameters.

#### 2.2.2. BE Assessment

Binge eating disorder was diagnosed according to the DSM-5 criteria [APA] [4]. These include the following: “A—Recurrent episodes of binge eating. An episode of binge eating is characterized by (A1) Eating, in a discrete period of time, an amount of food that is definitely larger than most people would eat in a similar period of time under similar circumstances. (A2) A sense of lack of control over eating during the episode (for example, a feeling that one cannot stop eating or control what or how much one is eating). B—The binge-eating episodes are associated with three (or more) of the following: (B1) Eating much more rapidly than normal. (B2) Eating until feeling uncomfortably full. (B3) Eating large amounts of food when not feeling physically hungry (B4). Eating alone because of feeling embarrassed by how much one is eating. (B5) Feeling disgusted with oneself, depressed, or very guilty afterwards. C—Marked distress regarding binge eating is present. D—The binge eating occurs, on average, at least once a week for three months. E—The binge eating is not associated with the recurrent use of inappropriate compensatory behavior (for example, purging) and does not occur exclusively during the course of anorexia nervosa, bulimia nervosa, or avoidant/restrictive food intake disorder”.

For a diagnosis to be made, criteria A1 and A2 must be present, with at least three or more of criteria B1 to B5. In terms of frequency, episodes must occur at least once a week over a period of at least three months. Episodes should not be associated with compensatory behaviors and are usually associated with distress over the episodes. To make a diagnosis using these criteria, a clinician needs about 20 min to interview the patient.

#### 2.2.3. Personality Assessment

To assess temperament and character, The *Temperament and Character Inventory* (TCI) by C. Robert Cloninger was used to provide a comprehensive biopsychosocial model of personality as it develops within individuals [17].

The TCI is based on a biopsychosocial model of complex interactions between genetic, psychological, social, cultural, and spiritual constructs.

C.R. Cloninger suggested that novelty seeking, harm avoidance, and reward dependence are correlated with low basal dopaminergic activity, high serotonergic activity, and low basal noradrenergic activity, respectively [18].

In our study we used the Polish adaptation of the TCI by E. Hornowska, [19] which is an operationalization of the psychobiological model of personality by C. R. Cloninger. It consists of 240 statements for which the respondent has to choose one of two possibilities—“true” or “false”. They consist of four dimensions of temperament and three dimensions of character, as shown in Table 1.

##### The Psychometric Properties of Polish Version of TCI

The reliability of the questionnaire for the Polish version is comparable to the original TCI and is expressed as internal consistency and absolute stability of the inventory. The reliability of the main dimensions of the test in the American version varies from 0.65 to 0.89. In the Polish version it is comparable and varies from 0.50 to 0.87. The values of the reliability coefficients for the main dimensions are presented in Table 2.

### 2.3. Statistical Analysis

Statistical analysis was performed using IBM SPSS Statistics for Windows, version 29 (IBM Corp., Armonk, NY, USA).

Quantitative variables were described by mean, standard deviation, median, first and third quartiles, and minimum and maximum values. For qualitative variables, frequencies and percentages of categories were reported. Appropriate statistical procedures were used to test the hypotheses.

The normality of the data was checked using the Shapiro–Wilk test. Only three empirical distributions were not normal (harm avoidance, persistence, cooperativeness).

The comparison of the group’s performance with accepted norms was carried out using a single-sample t-test. This test is used to verify the hypothesis that the test sample with a given mean comes from the population for which the mean is a given value.

The subgroups drawn from the total sample were compared using a non-parametric test (violation of the assumption of equality of groups [chi-square test] and violation of the assumption of equality of variance [Levene’s test]).

The Kruskal–Wallis H test was used. It is used to test the hypothesis of non-significance of differences between the medians of the variable under study in more than two populations (assuming that the distributions of the variable are close to each other). Significance values were adjusted using the Bonferroni method.

The results of the analysis were accepted as statistically significant at *p* <0.05.

## 3. Results

### 3.1. Descriptive Statistics for Subgroups

#### 3.1.1. PCOS Women Without Binge Eating Disorder

Descriptive statistics for the temperament of PCOS women without binge eating disorder are presented in supplementary materials in Table S1 by mean, standard deviation, minimum and maximum values, first and third quartile values, and median values for each temperament dimension and its subscales.

Descriptive statistics for the character of PCOS women without binge eating disorder are presented in supplementary materials in Table S2 by mean, standard deviation, minimum and maximum values, first and third quartile values, and median values for each temperament dimension and its subscales.

#### 3.1.2. PCOS Women with Binge Eating Disorder

Descriptive statistics for temperament dimensions for PCOS women with binge eating disorder are presented supplementary materials in Table S3 by mean, standard deviation, minimum and maximum values, first and third quartile values, and median values for each temperament dimension and its subscales.

Descriptive statistics for character dimensions for PCOS women with binge eating disorder are presented in supplementary materials in Table S4 by mean, standard deviation, minimum and maximum values, first and third quartile values, and median values for each temperament dimension and its subscales.

### 3.2. Comparison of Study Groups to the Normalization Group

The normalization group is the group of people whose test results are used to create norms. This group should be representative of the population to which the test is addressed. In the case of psychological tests, the normalization group should be appropriately diverse in terms of age, gender, education, and other relevant demographic factors. Its results are analyzed to determine what the typical results are for the population and what the differences are between the different groups. Adaptation of the TCI inventory to the Polish normalization group was carried out by Hornowska et al. [19].

Statistical analysis to compare the study groups with the normalization group was performed by using single-sample t-test. Means and standard deviations of the study group versus the normalization group were compared. The obtained results of the analysis were accepted as statistically significant at *p* < 0.05.

#### 3.2.1. PCOS Women Without Binge Eating Disorder

The main statistically significant differences on personality traits between the PCOS–non-BED group and the normalization group are presented in Table 3.

A comparison of the temperament of PCOS women without binge eating disorder with the normalization group is shown in Table 4. Patients in the PCOS group presented statistically significantly higher scores in the reward dependence dimension (16.49 vs. 15.33; *p* = 0.010), especially in the attachment subscale (5.98 vs. 5.16; *p* = 0.004), compared to the normalization group. There were also statistically significant higher scores in the persistence dimension in the PCOS group (4.96 vs. 4.09; *p* = 0.001).

A comparison of the character of PCOS women without binge eating disorder with the normalization group is shown in Table 5. Patients in the PCOS group present statistically significantly lower scores in the self-transcendence dimension (12.89 vs. 16.35; *p* < 0.001), especially in the transpersonal identification (2.94 vs. 3.61; *p* = 0.048) and spiritual acceptance (5.51 vs. 7.51; *p* < 0.001) subscales, in comparison with the normalization group.

#### 3.2.2. PCOS Women with Binge Eating Disorder

The main statistically significant differences on personality traits between the PCOS–BED group and the normalization group are presented in Table 6.

A comparison of the temperament of PCOS women with binge eating disorder and the normalization group is shown in Table 7. Patients in the PCOS–BED group present statistically significantly higher scores on the Harm Avoidance dimension (23.43 vs. 16.74; *p* < 0.001) in all subscales—anticipatory worry (7.13 vs. 4.97; *p* < 0.001), fear of uncertainty (4.98 vs. 4.37; *p* = 0.024), shyness (5.39 vs. 3.96; *p* < 0.001), and fatigability (5.93 vs. 3.62; *p* < 0.001). There were also statistically significantly higher scores on the reward dependence dimension (16.74 vs. 15.33; *p* = 0.003) in the study group, especially on the sentimentality subscale (7.61 vs. 6.96; *p* = 0.011).

A comparison of the character of PCOS women with binge eating disorder and the normalization group is presented in Table 8. Patients in the PCOS–BED group present statistically significantly lower scores on all subscales of the self-directedness dimension (18.87 vs. 26.78; *p* < 0.001)—responsibility (4.26 vs. 5.17; *p* = 0.004), purposeful (3.78 vs. 5.24; *p* < 0.001), resourcefulness (2.35 vs. 3.20; *p* < 0.001), self-acceptance (3.48 vs. 5.69; *p* < 0.001) and enlightened second nature (4.78 vs. 7.30; *p* < 0.001). Also, statistically significant lower scores on the compassion subscale of the cooperativeness dimension (6.93 vs. 7.70; *p* = 0.048) and lower scores on the spiritual acceptance subscale of the self-transcendence dimension (6.39 vs. 7.51; *p* = 0.015) were observed in the PCOS–BED group compared to the normalization group.

### 3.3. Cross-Group Comparison

The subgroups extracted from the entire sample were compared using a non-parametric test (violation of the assumption of equality of groups [chi-square test] and violation of the assumption of equality of variance [Levene’s test]).

The Kruskal–Wallis H-test was used. Significance values were adjusted using the Bonferroni method.

The results of the analysis were accepted as statistically significant at *p* <0.05.

The main statistically significant differences in personality traits between the PCOS and PCOS–BED group and their impact on binge eating behaviors are presented in Table 9.

The comparison of temperament between groups is shown in Table 10. Patients with PCOS–BED presented statistically significantly higher scores in the harm avoidance dimension (23.43 vs. 18.21; H = 16.497; *p* < 0.001), especially in anticipatory worry (7.13 vs. 5.15; H = 17.213; *p* < 0.001), shyness (5.39 vs. 3.85; H = 13.272; *p* = 0.001), and fatigability (5.93 vs. 4.32; H = 11.528; *p* = 0.003) subscales compared to PCOS patients. Statistically significant lower scores on the persistence dimension (4.04 vs. 4.96; H7.207; *p* = 0.027) were also observed in the PCOS–BED subgroup compared to the PCOS subgroup.

The Bonferroni post-hoc test for comparison of temperament between groups is shown in Table 11.

The comparison of character between the groups is shown in Table 12. Patients with PCOS–BED presented statistically significantly lower scores in the self-directedness dimension (18.87 vs. 26.66; H = 21.375; *p* < 0.001), especially in the purposefulness (3.78 vs. 5.21; H = 12.520; *p* = 0.002), resourcefulness (2.35 vs. 3.43; H = 10.200; *p* = 0.006), self-acceptance (3.48 vs. 5.79; H = 16.897; *p* < 0.001) and enlightened second nature subscales (4.78 vs. 7.34; H = 21.097; *p* < 0.001) compared to PCOS patients.

The Bonferroni post-hoc test for comparison of character between groups is shown in Table 13.

## 4. Discussion

To the best of the authors’ knowledge, this is the first study to assess temperament and personality in PCOS women with BED. There are studies in the literature that assess personality and its components in patients with eating disorders as well as in women with PCOS. However, to date, none of these have assessed personality components in individuals with both conditions and whether there is a specific personality trait that may predispose PCOS women to BED more than healthy women. Considering that the prevalence of BED in PCOS women is higher than in the general population of healthy women, and that the co-occurrence of both conditions may worsen an individual’s health status, influence treatment compliance, and require a different treatment approach including targeted psychotherapeutic interventions, the results of our study may shed new light on the perception of PCOS and contribute to a more effective therapeutic approach for this group of patients.

Our research has two strengths. First, it assesses personality using the TCI. This inventory is more reliable than other measures of personality and provides a reproducible assessment of inter-individual variability in personality traits in subjects without specific psychopathology [20]. Second, it is the first research to assess personality traits in PCOS patients with binge eating disorder.

Our research shows that PCOS women with BED have specific personality traits compared to PCOS women without BED. Their temperament is characterized by higher scores on the harm avoidance dimension and its three subscales—anticipatory worry, shyness, and fatigability—and lower scores on the persistence dimension. The character of PCOS women with BED compared to PCOS women without BED is characterized by lower scores in the self-directedness dimension and its four subscales—purposeful, resourcefulness, self-acceptance, and enlightened second nature.

Fassino, S. et al. point out in their research that all women with eating disorders are characterized by high harm avoidance and low self-directedness. The author emphasizes that these dimensions help to distinguish between women with eating disorders and healthy controls [21]. Comparable results are presented by Dalle Grave R. et al. in their research [15]. The authors state that patients with binge eating disorder had significantly higher scores on the dimensions of novelty seeking and harm avoidance. The presence of severe binge eating was positively associated with novelty seeking and harm avoidance, and negatively associated with self-directedness.

The results mentioned in previous research are mainly similar to ours. According to Cloninger C.R. et al., self-directedness measures internal organization and the ability to set and pursue meaningful goals [17]. Low scores on the self-directedness dimension are associated with immature behavior, self-humbling, and an inability to set and pursue goals.

Muller et al., in their research, point out that patients diagnosed with eating disorders tend to blame other people and situations for the frustrations they have to endure. They tend to believe that their behavior is mostly determined by influences beyond their control or against their will. As a result, they have poor willpower and are often unable to resist their temperamental impulses [22]. Difficulty with impulse control and weak willpower observed in PCOS–BED women in our study may favor the development of eating disorders in these individuals as well as hinder the treatment process.

Self-directedness was described by Bulik C.M. et al. as the only personality variable with a negative prognostic value for treatment efficacy. It predicted treatment outcome at a one-year follow-up in patients with bulimia nervosa treated with cognitive behavioral therapy [23]. This may explain why some women with PCOS find it more difficult to maintain a normal due weight, to comply with medical recommendations.

It shows that lower scores on the self-directedness dimension in PCOS women with BED may be associated with a higher risk of overweight and obesity, inappropriate food intake, lower ability to adhere to exercise recommendations, and treatment compliance. In addition, the associated character immaturity may influence the therapeutic relationship between doctor and patient. In our opinion, it can lead to situations where the patient blames external factors, including the alleged incompetence of the treating physician, for the lack of adequate response to therapy. This may suggest that this group of patients requires a specific therapeutic approach that takes into account the personal characteristics of the patient in order to achieve satisfactory results and long-term cooperation in the treatment process.

Harm avoidance is described by Cloninger C.R. et al. as a temperamental trait associated with the ability to cope with situations that may be perceived by an individual as harmful [20]. It describes the individual’s predisposition to respond to situations that may cause emotional distress with behavioral inhibition, fear, anxiety, and depression.

A high level of harm avoidance is usually found in depressive and anxiety disorders, as indicated in the literature [17,24,25,26,27]. It seems likely that greater severity of the above temperamental traits may be positively correlated with the occurrence of rumination, which in the cognitive-behavioral model is one of the elements that both sustains the current depressive episode and correlates with the risk of recurrent depressive episodes. The presence of anxiety symptoms and anxiety disorders also correlates positively with higher scores on the harm avoidance dimension [28,29].

The character trait that correlates with the severity of anxiety disorders, according to Cloninger et al., is the self-directedness dimension, and the direction of the correlation is negative [28].

The fact that PCOS patients with BED are characterized by greater harm avoidance than PCOS patients without BED may mean that this particular group of patients has a general temperamental predisposition that increases the risk of developing an eating disorder in response to stressors [30].

In our study we found that PCOS women with BED have higher scores on the anticipatory worry, shyness, and fatigue subscales of the harm avoidance dimension.

Cloninger R.C. et al. point out that people with high scores on the fatigability subscale may have less energy and a strong tendency to get tired compared to other people. They may also tend to become tired easily and recover slowly from stressors and minor illnesses [17]. According to some authors, this seems to correlate with a low serotoninergic tone [17,31].

All the above findings may predispose them to ruminative tendencies, experiencing anxiety and depressive mood as well as aggravation of binge eating symptoms.

Barberis N. et al. in their study indicate that PCOS women present a significant path from dysmorphic concerns to eating disorders [32]. The authors point out a statistically significant indirect association from BMI to eating disorders by dysmorphic concerns in PCOS women. This group of women is more likely to have problems losing weight due to the metabolic consequences of the disease and other comorbidities. According to the authors, these can promote maladaptive compensatory strategies used to control food intake and body size, such as eating disorders. In the long term, this can alter the way individuals function and lead to impaired social, physical, and psychological adjustment.

Fassino S. et al. point out that high harm avoidance and low self-directedness may be considered as the basic personality of the eating disorders spectrum [21].

Considering that almost one in two women in our study met the diagnosis of binge eating disorder and scored high on harm avoidance and low on self-directedness, this suggests that a significant percentage of PCOS patients have a personality trait that may predispose them to eating disorders. To date, there are no recommendations from scientific committees to assess temperament and personality in PCOS patients. We believe that a routine personality assessment should be carried out when PCOS is diagnosed. This would identify those PCOS women who may be predisposed to a more severe course of the disease, a higher risk of metabolic complications, eating disorders, and difficulties in complying with medical recommendations. As a result, new approaches to treatment strategies for this group of patients should be applied.

## 5. Conclusions

Women with PCOS have a higher risk of developing binge eating disorder compared to healthy controls. The group of PCOS women with co-occurrence of BED have specific personality traits that may predispose them to eating disorders. By screening PCOS patients for the presence of certain personality traits, we could identify individuals at risk. PCOS patients with BED may be at higher risk of health problems and treatment failure, and may require a different therapeutic approach to treatment, including psychotherapeutic interventions.

## Figures and Tables

**Table 1:** Structure of The Temperament and Character Inventory (TCI).

Personality	
Temperament	Character
**Novelty seeking (NS):** Exploratory excitability (NS1) Impulsiveness (NS2) Extravagance (NS3) Disorderliness (NS4)	**Self-directedness (SD):** Responsibility (SD1) Purposeful (SD2) Resourcefulness (SD3) Self-acceptance (SD4) Enlightened second nature (SD5)
**Harm avoidance (HA):** Anticipatory worry (HA1) Fear of uncertainty (HA2) Shyness (HA3) Fatigability (HA4)	**Cooperativeness (CO):** Social acceptance (C1) Empathy (C2) Helpfulness (C3) Compassion (C4) Pure-hearted conscience (C5)
**Reward dependence (RD):** Sentimentality (RD1) Attachment (RD2) Dependence (RD3)	**Self-transcendence (ST):** Self-forgetful (ST1) Transpersonal identification (ST2) Spiritual acceptance (ST3)
**Persistence (PS)**	

**Table 2:** The values of reliability coefficients of TCI main dimensions.

Personality	Dimensions	Reliability Coefficient
**Temperament**	Novelty seeking (NS) Harm avoidance (HA) Reward dependence (RD) Persistence (PE)	0.79 0.87 0.65 0.50
**Character**	Self-directedness (SD) Cooperativeness (CO) Self-transcendence (ST)	0.85 0.85 0.84

**Table 3:** The main differences on personality traits between PCOS–non-BED group and normalization group.

Personality	Normalization Group		PCOS Without BED		Statistics
	M	SD	M	SD	*p*
**Temperament**					
***Reward Dependence***	**15.33**	3.50	**16.49**	2.95	0.010 *
**Attachment**	**5.16**	1.87	**5.98**	1.86	0.004 *
***Persistence***	**4.09**	1.82	**4.96**	1.64	0.001 *
The high scores on *reward dependence* scale of the PCOS–non-BED group may indicate sensitivity to the looks and opinions of others and to social cues. Relationships with others are important to such individuals. They often value intimacy over privacy. They are very sensitive to rejection and neglect, which can contribute to feelings of increased psychological tension and predispose them to anxiety and depressive disorders.					
**Character**					
***Self-Transcendence***	**16.35**	6.08	**12.89**	6.13	<0.001 *
**Transpersonal Identification**	**3.61**	2.21	**2.94**	2.28	0.048 *
**Spiritual Acceptance**	**7.51**	2.95	**5.51**	2.94	<0.001 *
The lower scores on the *self-transcendence* scale of the PCOS–non-BED group may indicate impatience, lack of satisfaction with one’s own qualities, and difficulty in accepting discomfort and suffering. This can predispose them to body image dissatisfaction and psychological distress.					

Asterisk is used for statistically significant *p*-values. Bold shows values compared in a test between which are differences.

**Table 4:** Comparison of temperament of PCOS women without binge eating disorder to the normalization group.

Temperament	Normalization Group		PCOS Group		Significance of Differences
	M	SD	M	SD	
*NOVELTY SEEKING*	21.74	6.47	20.70	6.03	0.244
Exploratory excitability	6.0	2.30	6.13	2.37	0.714
Impulsiveness	4.88	2.45	4.32	2.29	0.101
Extravagance	6.10	2.23	5.79	2.13	0.318
Disorderliness	4.78	1.93	4.47	1.80	0.242
*HARM AVOIDANCE*	16.74	7.24	18.21	7.10	0.162
Anticipatory worry	4.97	2.75	5.15	2.47	0.621
Fear of uncertainty	4.37	2.08	4.89	2.16	0.103
Shyness	3.96	2.20	3.85	2.24	0.740
Fatigability	3.62	2.34	4.32	2.62	0.074
***REWARD DEPENDENCE***	**15.33**	3.50	**16.49**	2.95	0.010 *
Sentimentality	6.96	1.99	7.09	1.78	0.632
**Attachment**	**5.16**	1.87	**5.98**	1.86	0.004 *
Dependence	3.21	1.34	3.43	1.33	0.273
***PERSISTENCE***	**4.09**	1.82	**4.96**	1.64	0.001 *

*t*-test; * < 0.05.

**Table 5:** Comparison of character of PCOS women without binge eating disorder to the normalization group.

Character	Normalization Group		PCOS Group		Significance of Differences
	M	SD	M	SD	
*SELF-DIRECTEDNESS*	26.78	7.86	26.66	7.84	0.917
Responsibility	5.17	2.16	4.89	2.31	0.417
Purposeful	5.24	1.80	5.21	1.76	0.916
Resourcefulness	3.20	1.54	3.43	1.57	0.330
Self-acceptance	5.69	2.77	5.79	3.06	0.829
Enlightened second nature	7.30	2.61	7.34	2.45	0.910
*COOPERATIVENESS*	32.49	5.92	33.30	4.48	0.176
Social acceptance	6.80	1.45	6.94	1.22	0.449
Empathy	4.83	1.28	5.15	1.20	0.074
Helpfulness	5.89	1.44	6.17	1.15	0.101
Compassion	7.70	2.53	7.81	2.31	0.749
Pure-hearted conscience	7.27	1.55	7.23	1.16	0.833
***SELF-TRANSCENDENCE***	**16.35**	6.08	**12.89**	6.13	<0.001 *
Self-forgetful	5.14	2.56	4.47	2.50	0.072
**Transpersonal identification**	**3.61**	2.21	**2.94**	2.28	0.048 *
**Spiritual acceptance**	**7.51**	2.95	**5.51**	2.94	<0.001 *

*t*-test; * < 0.05.

**Table 6:** The main differences on personality traits between PCOS–BED group and normalization group.

Personality	Normalization Group		PCOS–BED Group		Statistics
	M	SD	M	SD	
**TEMPERAMENT**					
***HARM AVOIDANCE***	**16.74**	7.24	**23.43**	7.37	<0.001 *
**Anticipatory worry**	**4.97**	2.75	**7.13**	2.76	<0.001 *
**Fear of uncertainty**	**4.37**	2.08	**4.98**	1.93	0.024 *
**Shyness**	**3.96**	2.20	**5.39**	2.26	<0.001 *
**Fatigability**	**3.62**	2.34	**5.93**	2.43	<0.001 *
***REWARD DEPENDENCE***	**15.33**	3.50	**16.74**	3.27	0.003 *
**Sentimentality**	**6.96**	1.99	**7.61**	1.82	0.011 *
**CHARACTER**					
***SELF-DIRECTEDNESS***	**26.78**	7.86	**18.87**	7.85	<0.001 *
**Responsibility**	**5.17**	2.16	**4.26**	2.24	0.004 *
**Purposeful**	**5.24**	1.80	**3.78**	2.09	<0.001 *
**Resourcefulness**	**3.20**	1.54	**2.35**	1.62	<0.001 *
**Self-acceptance**	**5.69**	2.77	**3.48**	2.12	<0.001 *
**Enlightened second nature**	**7.30**	2.61	**4.78**	2.89	<0.001 *
*COOPERATIVENESS*					
**Compassion**	**7.70**	2.53	**6.93**	2.81	0.048 *
*SELF-TRANSCENDENCE*					
**Spiritual acceptance**	**7.51**	2.95	**6.39**	3.27	0.015 *
The high *harm avoidance* and *reward dependence* as well as low *self-directedness*, *compassion,* and *spiritual acceptance* of the PCOS–BED group may reflect a tendency towards anxiety and avoidance behaviors that could exacerbate BED symptoms.					

Asterisk is for marking statistically significant *p* values. Bold shows between which parameters are differences.

**Table 7:** Comparison of temperament of PCOS women with binge eating disorder to the normalization group.

Temperament	Normalization Group		PCOS–BED Group		Significance of Differences
	M	SD	M	SD	
*NOVELTY SEEKING*	21.74	6.47	20.83	6.98	0.440
Exploratory excitability	6.0	2.30	5.69	2.63	0.384
Impulsiveness	4.88	2.45	4.70	2.23	0.564
Extravagance	6.10	2.23	5.85	2.59	0.484
Disorderliness	4.78	1.93	4.59	1.90	0.471
***HARM AVOIDANCE***	**16.74**	7.24	**23.43**	7.37	<0.001 *
**Anticipatory worry**	**4.97**	2.75	**7.13**	2.76	<0.001 *
**Fear of uncertainty**	**4.37**	2.08	**4.98**	1.93	0.024 *
**Shyness**	**3.96**	2.20	**5.39**	2.26	<0.001 *
**Fatigability**	**3.62**	2.34	**5.93**	2.43	<0.001 *
***REWARD DEPENDENCE***	**15.33**	3.50	**16.74**	3.27	0.003 *
**Sentimentality**	**6.96**	1.99	**7.61**	1.82	0.011 *
Attachment	5.16	1.87	5.65	2.09	0.092
Dependence	3.21	1.34	3.48	1.38	0.155
***PERSISTENCE***	**4.09**	1.82	**4.04**	1.87	0.836

*t*-test; * < 0.05.

**Table 8:** Comparison of character of PCOS women with binge eating disorder to the normalization group.

Character	Normalization Group		PCOS–BED Group		Significance of Differences
	M	SD	M	SD	
***SELF-DIRECTEDNESS***	**26.78**	7.86	**18.87**	7.85	<0.001 *
**Responsibility**	**5.17**	2.16	**4.26**	2.24	0.004 *
**Purposeful**	**5.24**	1.80	**3.78**	2.09	<0.001 *
**Resourcefulness**	**3.20**	1.54	**2.35**	1.62	<0.001 *
**Self-acceptance**	**5.69**	2.77	**3.48**	2.12	<0.001 *
**Enlightened second nature**	**7.30**	2.61	**4.78**	2.89	<0.001 *
*COOPERATIVENESS*	32.49	5.92	31.11	6.81	0.170
Social acceptance	6.80	1.45	6.70	1.60	0.660
Empathy	4.83	1.28	4.81	1.48	0.940
Helpfulness	5.89	1.44	5.80	1.25	0.584
**Compassion**	**7.70**	2.53	**6.93**	2.81	0.048 *
Pure-hearted conscience	7.27	1.55	6.87	1.53	0.060
*SELF-TRANSCENDENCE*	16.35	6.08	14.94	6.24	0.104
Self-forgetful	5.14	2.56	5.30	2.19	0.603
Transpersonal identification	3.61	2.21	3.26	2.30	0.268
**Spiritual acceptance**	**7.51**	2.95	**6.39**	3.27	0.015 *

*t*-test; * < 0.05.

**Table 9:** The main differences in personality traits between PCOS and PCOS–BED.

Personality	PCOS Without BED		PCOS with BED		Normalization Group		Statistics	
	M	SD	M	SD	M	SD	H	*p*
**TEMPERAMENT**								
***HARM AVOIDANCE***	**18.21**	7.10	**23.43**	7.37	16.74	7.24	**16.497**	<0.001 *
**Anticipatory worry**	**5.15**	2.47	**7.13**	2.76	4.97	2.75	**17.213**	<0.001 *
**Shyness**	**3.85**	2.24	**5.39**	2.26	3.96	2.20	**13.272**	0.001 *
**Fatigability**	**4.32**	2.62	**5.93**	2.43	3.62	2.34	**11.528**	0.003 *
***PERSISTENCE***	**4.96**	1.64	**4.04**	1.87	4.09	1.82	**7.207**	0.027 *
The high *harm avoidance* of the PCOS–BED group may reflect a tendency towards anxiety and avoidance behaviors that could exacerbate BED symptoms. The high scores on anticipatory worry of the PCOS–BED group may be reflected in a tendency to worry excessively and difficulty recovering from subjectively distressing events, which may promote compulsive ways of regulating emotions and binge eating. The high scores on the *shyness* subscale of the PCOS–BED group may be associated with greater perceived social anxiety and difficulty adapting to new situations. Consequently, the lower levels of social support experienced by these individuals may predispose them to alternative forms of reward and reduction of perceived emotional distress through compulsive behaviors such as binge eating. The high scores on the *fatigability* subscale of the PCOS–BED group may be associated with lower perceived levels of vital energy. This predisposes them to rapid fatigue and the need for extra rest, and slower recovery from subjectively stressful situations. This may be associated with a reluctance to engage in physical activity and less consistent performance. The above can lead to obesity and body image dissatisfaction and be a trigger for emotional eating. The lower scores on the *persistence* scale of the PCOS–BED group may be manifested by a tendency to give up when faced with difficulties, setbacks, obstacles or criticism. This may be reflected in poorer therapeutic cooperation, inconsistency in treatment, and lower treatment effectiveness.								
**CHARACTER**								
***SELF-DIRECTEDNESS***	**26.66**	7.84	**18.87**	7.85	26.78	7.86	**21.375**	<0.001 *
**Purposeful**	**5.21**	1.76	**3.78**	2.09	5.24	1.80	**12.520**	0.002 *
**Resourcefulness**	**3.43**	1.57	**2.35**	1.62	3.20	1.54	**10.200**	0.006 *
**Self-acceptance**	**5.79**	3.06	**3.48**	2.12	5.69	2.77	**16.897**	<0.001 *
**Enlightened second nature**	**7.34**	2.45	**4.78**	2.89	7.30	2.61	**21.097**	<0.001 *
The lower scores on the *self-directedness* scale of the PCOS–BED group can manifest themselves as shifting responsibility, less efficiency, and irresponsibility. The behavior of such individuals may be driven more by responses to stimuli and external pressures than by their own goals and values. Such a constellation of characteristics may predispose them to eating disorders and may also be a predictor of poorer response to treatment. The lower scores on the *purposeful* subscale of the PCOS–BED group may manifest as difficulties in delaying gratification. This may predispose them to the development of compulsive-type disorders, including binge eating. It may also indicate difficulty in adhering to treatment recommendations. The lower scores on the *resourcefulness* subscale of the PCOS–BED group can manifest in feelings of low self-confidence, incompetence, and poor problem-solving skills. This can increase emotional distress and the risk of developing an eating disorder. The lower scores on the *self-acceptance* subscale may be characterized by low self-esteem and a lack of acceptance of one’s mental and physical characteristics, which increases anxiety. The above may predispose them to a higher risk of developing an eating disorder. The lower scores on the *enlightened second nature* subscale can be characterized by inconsistency in action and a lack of consistency and strong will. They fail to resist strong temptations, despite being aware of the negative consequences of their actions. These characteristics may be highly predictive of both the onset and duration of eating disorders.								

Asterisk and bold values as below. It also ameliorate visibility of the results.

**Table 10:** Cross-group comparison of temperament.

Temperament	Group 1		Group 2		Group 3		Statistics	
	M	SD	M	SD	M	SD	H	*p*
*NOVELTY SEEKING*	20.70	6.03	22.75	6.29	20.83	6.98	1.790	0.409
Exploratory excitability	6.13	2.37	6.63	2.83	5.69	2.63	2.401	0.301
Impulsiveness	4.32	2.29	4.33	2.32	4.70	2.23	0.602	0.740
Extravagance	5.79	2.13	6.38	2.18	5.85	2.59	1.210	0.546
Disorderliness	4.47	1.80	5.42	2.39	4.59	1.90	2.460	0.292
***HARM AVOIDANCE***	**18.21**	7.10	17.75	7.11	**23.43**	7.37	**16.497**	<0.001 *
**Anticipatory worry**	**5.15**	2.47	5.08	2.38	**7.13**	2.76	**17.213**	<0.001 *
Fear of uncertainty	4.89	2.16	4.42	2.02	4.98	1.93	1.644	0.440
**Shyness**	**3.85**	2.24	3.88	2.25	**5.39**	2.26	**13.272**	0.001 *
**Fatigability**	**4.32**	2.62	4.38	2.65	**5.93**	2.43	**11.528**	0.003 *
*REWARD DEPENDENCE*	16.49	2.95	17.04	3.03	16.74	3.27	0.392	0.822
Sentimentality	7.09	1.78	6.88	1.83	7.61	1.82	4.115	0.128
Attachment	5.98	1.86	6.33	1.71	5.65	2.09	1.769	0.413
Dependence	3.43	1.33	3.83	1.46	3.48	1.38	1.368	0.505
***PERSISTENCE***	**4.96**	1.64	4.13	1.85	**4.04**	1.87	**7.207**	0.027 *

Kruskal–Wallis H test; * < 0.05; Group 1—[PCOS]—PCOS women without binge eating disorder; Group 2—[PCOS–non-BED]—PCOS women who did not meet the diagnosis of binge eating disorder but answered positively for at least one question when evaluated for DSM-5 criteria of binge eating; Group 3—[PCOS–BED]—PCOS women with diagnosis of binge eating disorder.

**Table 11:** Post-hoc test of cross-group comparison of temperament.

Temperament	Significance of Differences		Multiple Comparisons
	H	*p*	
***HARM AVOIDANCE***	**16.497**	<0.001 *	**1–3; 2–3**
**Anticipatory worry**	**17.213**	<0.001 *	**1–3; 2–3**
**Shyness**	**13.272**	0.001 *	**1–3; 2–3**
**Fatigability**	**11.528**	0.003 *	**1–3; 2–3**
***PERSISTENCE***	**7.207**	0.027 *	**1–3**

Significance values were adjusted using the Bonferroni method. Asterisk is used for statistically significant *p*-values. Bold shows values compared in a test between which are differences.

**Table 12:** Cross-group comparison of character.

Character	Group 1		Group 2		Group 3		Statistics	
	M	SD	M	SD	M	SD	H	*p*
***SELF-DIRECTEDNESS***	**26.66**	7.84	24.58	8.58	**18.87**	7.85	**21.375**	<0.001 *
Responsibility	4.89	2.31	5.46	1.91	4.26	2.24	4.872	0.087
**Purposeful**	**5.21**	1.76	4.79	1.69	**3.78**	2.09	**12.520**	0.002 *
**Resourcefulness**	**3.43**	1.57	2.83	1.74	**2.35**	1.62	**10.200**	0.006 *
**Self-acceptance**	**5.79**	3.06	5.63	3.21	**3.48**	2.12	**16.897**	<0.001 *
**Enlightened second nature**	**7.34**	2.45	5.88	3.10	**4.78**	2.89	**21.097**	<0.001 *
*COOPERATIVENESS*	33.30	4.48	33.54	4.72	31.11	6.81	2.268	0.322
Social acceptance	6.94	1.22	6.88	1.19	6.70	1.60	0.340	0.844
Empathy	5.15	1.20	5.58	1.02	4.81	1.48	4.808	0.090
Helpfulness	6.17	1.15	6.17	1.05	5.80	1.25	2.513	0.285
Compassion	7.81	2.31	7.92	2.32	6.93	2.81	3.227	0.199
Pure-hearted conscience	7.23	1.16	7.00	1.53	6.87	1.53	0.752	0.687
*SELF-TRANSCENDENCE*	12.89	6.13	14.67	6.67	14.94	6.24	2.979	0.226
Self-forgetful	4.47	2.50	4.79	2.08	5.30	2.19	3.210	0.201
Transpersonal identification	2.94	2.28	3.38	2.22	3.26	2.30	0.936	0.626
Spiritual acceptance	5.51	2.94	6.50	3.66	6.39	3.27	2.453	0.293

Kruskal–Wallis H test; * < 0.05; Group 1—[PCOS]—PCOS women without binge eating disorder; Group 2—[PCOS–non-BED]—PCOS women who did not meet the diagnosis of binge eating disorder but answered positively for at least one question when evaluated for DSM-5 criteria of binge eating; Group 3—[PCOS–BED]—PCOS women with diagnosis of binge eating disorder.

**Table 13:** Post-hoc test of cross-group comparison of character.

Character	Significance of Differences		Multiple Comparisons
	H	*p*	
***SELF-DIRECTEDNESS***	**21.375**	<0.001 *	**1–3; 2–3**
**Purposeful**	**12.520**	0.002 *	**1–3**
**Resourcefulness**	**10.200**	0.006 *	**1–3**
**Self-acceptance**	**16.897**	<0.001 *	**1–3; 2–3**
**Enlightened second nature**	**21.097**	<0.001 *	**1–3**

Significance values were adjusted using the Bonferroni method. Asterisk is used for statistically significant *p*-values. Bold shows values compared in a test between which are differences.

## Data Availability

The data presented in this study are available on request from the corresponding author. The data are not publicly available due to current calculations to the next publication from this study.

## Supplementary Materials

The following supporting information can be downloaded at: https://www.mdpi.com/article/10.3390/jcm13237100/s1, Table S1. Description of polycystic ovary syndrome criteria used in the study; Table S2: Descriptive statistics for temperament dimension for PCOS women without binge eating disorder; Table S3: Descriptive statistics for character dimension for PCOS women without binge eating disorder; Table S4: Descriptive statistics for temperament dimension for PCOS women with binge eating disorder; Table S5: Descriptive statistics for character dimension for PCOS women with binge eating disorder.
