# Supplementary material for: Temperamental and Character Traits as Risk Factors for Binge Eating Disorder in Women with Polycystic Ovary Syndrome
Source: J Clin Med. 2024 Nov 24;13(23):7100. doi: 10.3390/jcm13237100 (PMC11642575; doi:10.3390/jcm13237100)
Supplement: Supplementary file 1 [file jcm-13-07100-s001.zip › jcm-3315892-supplementary.pdf]

Journal of
Clinical Medicine

MDPI

Supplementary Materials:

Table S1. Description of polycystic ovary syndrome criteria used in the study.

PCOS criterion	Description
HYPERANDROGENISM	Clinical hyperandrogenism consists of hirsutism, acne and/or alopecia. The degree of hirsutism was assessed using the Ferriman-Gallwey score. A score of 4 or more con-firmed hirsutism. Biochemical hyperandrogenism consists of elevated levels of andro-gen hormones (testosterone and/or androstenedione).
ANOVULATORY MENSTRUAL CYCLES and/or INREQUENT OVULATIONS	Anovulation was confirmed by oligomenorrhoea and serum progesterone levels on days 22 to 24 of the cycle. A level of 3 ng/ml or less confirmed an anovulatory cycle. Oligomenorrhoea was defined as a menstrual cycle lasting more than 35 days, and secondary amenorrhoea was defined as the absence of menstrual bleeding for more than 6 months.
CHARACTERISTIC OVARIAN STRUCTURE ON ULTRASOUND	Pelvic ultrasound was performed using an Aloka 7 alpha device (Hita-chi-Aloka Medical America Inc., Wallingford, CT, USA) to assess ovarian morphology. PCOM was defined as the presence of follicle number per ovary (FNPO) ⩾ 20 in at least one ovary or follicle number per section (FNPS) ⩾ 10 in at least one ovary and/or ovarian volume > 10 ml in the absence of confounding pathology.

Table S2. Descriptive statistics for temperament dimension for PCOS women without binge eating disorder.

TEMPERAMENT	M	SD	Min	Max	Q1	Me	Q3
NOVELTY SEEKING	20.70	6.03	9.00	30.00	16.00	21.00	26.00
Exploratory excitability	6.13	2.37	0.00	11.00	5.00	6.00	8.00
Impulsiveness	4.32	2.29	0.00	9.00	3.00	4.00	6.00
Extravagance	5.79	2.13	1.00	9.00	4.00	6.00	8.00
Disorderliness	4.47	1.80	0.00	8.00	4.00	4.00	6.00
HARM AVOIDANCE	18.21	7.10	6.00	32.00	12.00	17.00	25.00
Anticipatory worry	5.15	2.47	1.00	10.00	3.00	5.00	7.00
Fear of uncertainty	4.89	2.16	0.00	7.00	3.00	5.00	7.00
Shyness	3.85	2.24	0.00	8.00	2.00	4.00	6.00
Fatigability	4.32	2.62	0.00	9.00	2.00	4.00	7.00
REWARD DEPENDENCE	16.49	2.95	9.00	22.00	14.00	17.00	19.00
Sentimentality	7.09	1.78	2.00	10.00	6.00	7.00	8.00
Attachment	5.98	1.86	2.00	8.00	4.00	7.00	8.00
Dependence	3.43	1.33	1.00	6.00	2.00	3.00	5.00
PERSISTENCE	4.96	1.64	2.00	8.00	4.00	5.00	6.00

M- mean, SD- standard deviation, Min– minimum value, Max– maximum value, Q1 – first quartile, Me- median, Q3 – third quartile

Table S3. Descriptive statistics for character dimension for PCOS women without binge eating disorder.

CHARACTER	M	SD	Min	Max	Q1	Me	Q3
SELF-DIRECTEDNESS	26.66	7.84	11.00	42.00	21.00	26.00	32.00
Responsibility	4.89	2.31	0.00	8.00	3.00	5.00	7.00
Purposeful	5.21	1.76	1.00	8.00	4.00	6.00	6.00
Resourcefulness	3.43	1.57	0.00	5.00	2.00	4.00	5.00
Self-acceptance	5.79	3.06	1.00	11.00	3.00	6.00	8.00
Enlightened second nature	7.34	2.45	2.00	12.00	6.00	8.00	9.00
COOPERATIVENESS	33.30	4.48	20.00	40.00	31.00	34.00	36.00
Social acceptance	6.94	1.22	3.00	8.00	7.00	7.00	8.00
Empathy	5.15	1.20	3.00	7.00	4.00	5.00	6.00
Helpfulness	6.17	1.15	3.00	8.00	5.00	6.00	7.00
Compassion	7.81	2.31	1.00	10.00	6.00	8.00	10.00
Pure-hearted conscience	7.23	1.16	5.00	9.00	6.00	7.00	8.00
SELF-TRANSENDENCE	12.89	6.13	3.00	26.00	8.00	12.00	19.00
Self-forgetful	4.47	2.50	1.00	9.00	2.00	4.00	7.00
Transpersonal identification	2.94	2.28	0.00	8.00	1.00	3.00	5.00
Spiritual acceptance	5.51	2.94	0.00	12.00	3.00	5.00	8.00

M- mean, SD- standard deviation, Min– minimum value, Max– maximum value, Q1 – first quartile, Me- median, Q3 – third quartile

Table S4. Descriptive statistics for temperament dimension for PCOS women with binge eating disorder.

TEMPERAMENT	M	SD	Min	Max	Q1	Me	Q3
NOVELTY SEEKING	20.83	6.98	6.00	38.00	16.00	21.50	25.25
Exploratory excitability	5.69	2.63	0.00	10.00	3.75	5.50	8.00
Impulsiveness	4.70	2.23	1.00	10.00	3.00	4.00	6.00
Extravagance	5.85	2.59	0.00	9.00	4.00	7.00	8.00
Disorderliness	4.59	1.90	1.00	9.00	3.00	4.00	6.00
HARM AVOIDANCE	23.43	7.37	2.00	34.00	20.00	23.50	29.00
Anticipatory worry	7.13	2.76	0.00	10.00	4.75	8.00	9.00
Fear of uncertainty	4.98	1.93	0.00	7.00	4.00	5.50	7.00
Shyness	5.39	2.26	0.00	8.00	4.00	6.00	7.25
Fatigability	5.93	2.43	0.00	9.00	4.00	6.00	8.00
REWARD DEPENDENCE	16.74	3.27	10.00	23.00	14.00	17.00	19.00
Sentimentality	7.61	1.82	3.00	10.00	7.00	8.00	9.00
Attachment	5.65	2.09	0.00	8.00	5.00	6.00	7.00
Dependence	3.48	1.38	1.00	6.00	2.00	3.00	5.00
PERSISTENCE	4.04	1.87	1.00	8.00	3.00	4.00	5.25

M- mean, SD- standard deviation, Min– minimum value, Max– maximum value, Q1 – first quartile, Me- median, Q3 – third quartile

Table S5. Descriptive statistics for character dimension for PCOS women with binge eating disorder.

CHARAKTER	M	SD	Min	Max	Q1	Me	Q3
SELF-DIRECTEDNESS	18.87	7.85	4.00	41.00	13.00	18.00	23.50
Responsibility	4.26	2.24	0.00	8.00	2.00	4.00	6.00
Purposeful	3.78	2.09	0.00	8.00	2.00	4.00	5.00
Resourcefulness	2.35	1.62	0.00	5.00	1.00	2.00	4.00
Self-acceptance	3.48	2.12	0.00	9.00	2.00	3.00	5.00
Enlightened second nature	4.78	2.89	0.00	12.00	3.00	5.00	6.00
COOPERATIVENESS	31.11	6.81	13.00	40.00	26.75	32.50	36.00
Social acceptance	6.70	1.60	1.00	8.00	6.00	7.00	8.00
Empathy	4.81	1.48	1.00	7.00	4.00	5.00	6.00
Helpfulness	5.80	1.25	3.00	8.00	5.00	6.00	7.00
Compassion	6.93	2.81	1.00	10.00	4.00	8.00	9.00
Pure-hearted conscience	6.87	1.53	3.00	9.00	6.00	7.00	8.00
SELF-TRANSENDENCE	14.94	6.24	1.00	30.00	10.00	15.00	18.00
Self-forgetful	5.30	2.19	1.00	10.00	3.75	6.00	7.00
Transpersonal identification	3.26	2.30	0.00	9.00	1.00	3.00	5.00
Spiritual acceptance	6.39	3.27	0.00	12.00	3.00	7.00	9.00

M- mean, SD- standard deviation, Min– minimum value, Max– maximum value, Q1 – first quartile, Me- median, Q3 – third quartile
